# A High-Throughput Study of the Electronic Structure and Physical Properties of Short-Period (GaAs)_m_(AlAs)_n_ (m, n ≤ 10) Superlattices Based on Density Functional Theory Calculations

**DOI:** 10.3390/nano8090709

**Published:** 2018-09-10

**Authors:** Qing-Lu Liu, Zong-Yan Zhao, Jian-Hong Yi, Zi-Yang Zhang

**Affiliations:** 1 Faculty of Materials Science and Engineering, Kunming University of Science and Technology, Kunming 650093, China; qluliu@kmust.edu.cn (Q.-L.L.); yijianhong@kmust.edu.cn (J.-H.Y.); 2 Key Laboratory of Nanodevices and Applications, Suzhou Institute of Nano-Tech and Nano-Bionics, Chinese Academy of Sciences, Suzhou 215123, China; zyzhang2014@sinano.ac.cn

**Keywords:** superlattices, high-throughput study, density functional theory calculations, electronic structure

## Abstract

As important functional materials, the electronic structure and physical properties of (GaAs)_m_(AlAs)_n_ superlattices (SLs) have been extensively studied. However, due to limitations of computational methods and computational resources, it is sometimes difficult to thoroughly understand how and why the modification of their structural parameters affects their electronic structure and physical properties. In this article, a high-throughput study based on density functional theory calculations has been carried out to obtain detailed information and to further provide the underlying intrinsic mechanisms. The band gap variations of (GaAs)_m_(AlAs)_n_ superlattices have been systematically investigated and summarized. They are very consistent with the available reported experimental measurements. Furthermore, the direct-to-indirect-gap transition of (GaAs)_m_(AlAs)_n_ superlattices has been predicted and explained. For certain thicknesses of the GaAs well (m), the band gap value of (GaAs)_m_(AlAs)_n_ SLs exponentially increases (increasing n), while for certain thicknesses of the AlAs barrier (n), the band gap value of (GaAs)_m_(AlAs)_n_ SLs exponentially decreases (increasing m). In both cases, the band gap values converge to certain values. Furthermore, owing to the energy eigenvalues at different k-points showing different variation trends, (GaAs)_m_(AlAs)_n_ SLs transform from a Γ-Γ direct band gap to Γ-M indirect band gap when the AlAs barrier is thick enough. The intrinsic reason for these variations is that the contributions and positions of the electronic states of the GaAs well and the AlAs barrier change under altered thickness conditions. Moreover, we have found that the binding energy can be used as a detector to estimate the band gap value in the design of (GaAs)_m_(AlAs)_n_ devices. Our findings are useful for the design of novel (GaAs)_m_(AlAs)_n_ superlattices-based optoelectronic devices.

## 1. Introduction

The artificial semiconductor superlattice (SL) is a specific form of layered fine composite, in which the nanoscale thin layers with different band gaps are alternately grown by the strict periods. It has been widely used in different application fields, such as quantum cascade lasers, high-frequency oscillators, solar cells, photodetectors, light emitting diodes, and so on [1,2,3,4], due to its new physical phenomena and mechanisms, such as quantum confinement, quantum hall effect, Brillouin-zone folding, and direct-to-indirect-gap transition [5]. The structure and properties of SLs are easily associated with coupled multiple quantum well systems. Along the growth direction, the lattice constant of SLs is much larger than the lattice constant of the components, so the original Brillouin-zone is subdivided into smaller zones in which the so-called sub-bands or folded bands are formed. On the other hand, because the potential barrier/well layers are nanoscale thin layers, there is a certain coupling interaction between the adjacent barriers/wells, which makes the separated energy levels expand into micro-bands. The properties of the sub-band and micro-band are determined by the structural parameters of SLs (such as components of the SL, depth and width of the potential well, height and width of the potential barrier, etc.), and determine the application performances of SLs. Therefore, these structural parameters are the key factors to designing SLs.

As representatives of compound semiconductors, III-V group materials have been used to construct SLs and are widely used in the field of nanophotonics. In our previous work, we investigated the effects of modulation p-doping on the thermal stability of InAs/GaAs quantum dot superluminescent diodes, confirming that the improved thermal stability is related to the built-in holes in quantum dot structures [6]. It is noted that gallium arsenide (GaAs) and aluminum arsenide (AlAs) are perfectly lattice-matched, and few difficulties are expected in the growth of type-I (GaAs)_m_(AlAs)_n_ semiconductor SLs, which consist of m monolayers of a GaAs well alternating with n monolayers of an AlAs barrier [7,8]. Many articles have been devoted to investigating the preparation, properties, and performances of (GaAs)_m_(AlAs)_n_ SLs [9,10]. Owing to its rich and important properties, (GaAs)_m_(AlAs)_n_ SLs are often used as the prototype to study the underlying intrinsic mechanisms [10,11,12,13]. Recently, Jiang et al. found Ga and Al atoms in GaAs/AlAs SLs are more susceptible to the radiation than those in the bulk AlAs and GaAs, in which the created defects have a profound effect on the electronic properties of GaAs/AlAs SLs (even metallicity are induced in some cases) [14]. Barkissy et al. reported the band gap of GaAs/AlAs SLs performed in the envelope function formalism with respect of thickness ratio [15].

However, due to the limitation of computational methods and computational resources, most early studies are limited to studying only a limited number of (GaAs)_m_(AlAs)_n_ SLs (for example m = n) [16,17,18]. Usually, conventional density functional theory (DFT) calculations only complete a limited number of computing tasks and need to be manually monitored at any time. Therefore, it is difficult to thoroughly understand the variation trend and mechanism of the electronic structure of (GaAs)_m_(AlAs)_n_ SLs with their structural parameters. At present, the high-throughput study based on DFT calculations allows carrying out large quantities of calculation and data screening, promotes the understanding of material properties and the construction of the material genome database, and provides useful information for the design of novel materials and devices. In fact, high-throughput computational methods represent a powerful tool for exploring materials space and for screening materials without having to synthesize them first [19]. Thus, in this article, it was adopted to study the electronic structure of short-period (GaAs)_m_(AlAs)_n_ (m, n ≤ 10) SLs in order to obtain detailed information and further provide the underlying intrinsic mechanisms, paying particular attention to the band gap variation of (GaAs)_m_(AlAs)_n_ SLs as a function of the periodic numbers m and n. By systematically analyzing the electronic structure and properties of 100 (GaAs)_m_(AlAs)_n_ SL models, the varying trends and laws of the electronic structure and properties of (GaAs)_m_(AlAs)_n_ SLs can be comprehensively understood. Moreover, the data and findings in this article will provide theoretical guidance and technical support for the development of III-V group semiconductor SLs based optoelectronic devices.

## 2. Computational Methods and Models

All of the calculations were performed using the periodic DFT package of Cambridge Serial Total Energy Package (CASTEP) codes [20]. All calculations were performed in the framework of generalized gradient approximation (GGA), using the Perdew–Burke–Ernzerhof exchange correlation potential for solids (PBEsol) [21,22]. According to the conventional DFT calculation method, all calculations in the present work were performed under the condition of absolute temperature 0 K. The energy cutoff for the plane wave basis was set as 340 eV. In order to get an accurate electronic structure, the method of GGA + U was adopted to overcome the well-known shortcoming of GGA [23]. Utilizing the GGA + U method, accurate band gaps could be obtained that could be compared with experimental measurements, as well as keeping the main features of the electronic structure obtained using standard DFT calculations. In the present work, the U value was set as 6.0 eV for all p-shells of GaAs layers, and 8.0 eV for p-shells of the AlAs layers. The Monkhorst–Pack scheme of K–points grid sampling was set as 4 × 4 × 4 for the irreducible Brillouin zone. The minimization algorithm chosen was the Broyden–Fletcher–Goldfarb–Shanno (BFGS) scheme [24]. Its convergence criteria were set as follows: the force on the atoms was less than 0.03 eV/Å, the stress on the atoms was less than 0.05 GPa, the atomic displacement was less than 0.001 Å, and the energy change per atom was less than 1.0 × 10^−5^ eV. Based on the optimized crystal structure, the electronic structure was then calculated. By these methods and settings, the reliable lattice parameters and electronic structure of GaAs and AlAs could be obtained, which were completely consistent with recent results in published articles.

**Figure 1:**
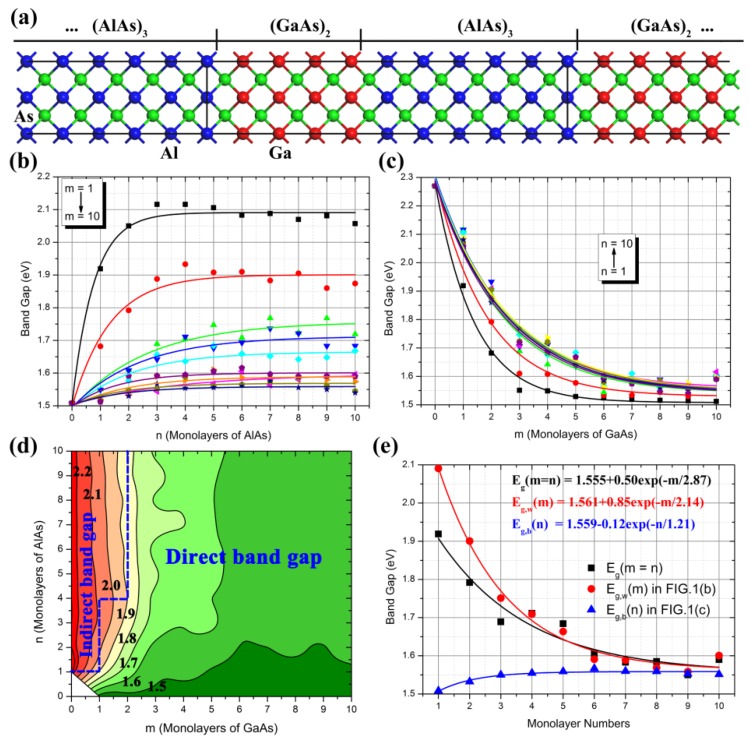
(**a**) The model of (GaAs)_m_(AlAs)_n_ SLs, in which the red, blue, and green spheres represent the Ga, Al, and As atoms, respectively; (**b**) the band gap values of (GaAs)_m_(AlAs)_n_ SLs as a function of monolayers of the AlAs barrier; (**c**) the band gap values of (GaAs)_m_(AlAs)_n_ SLs as function of the number of monolayers of GaAs well; (**d**) the contour map of band gap values of (GaAs)_m_(AlAs)_n_ SLs; (**e**) the special band gap values of (GaAs)_m_(AlAs)_n_ SLs as function of monolayers of GaAs well and/or AlAs barrier.

A superlattice is a periodic structure of layers of two (or more) materials. Typically, the thickness of one layer is several nanometers, which can also serve as a lower-dimensional quantum structure. In the present work, GaAs acts as a quantum well, while AlAs acts as a quantum barrier. The (GaAs)_m_(AlAs)_n_ SLs were constructed along on the [100] direction of the sphalerite GaAs or AlAs crystal structure. The subscript m or n denotes the period number along this direction. One period number refers to one periodic bulk (GaAs: 5.698 Å; AlAs: 5.720 Å), which equals to one monolayer. This setting is different to the settings in some published articles, in which one period equals to one molecular layer (GaAs: 2.849 Å; AlAs: 2.860 Å). However, the latter setting changes the symmetry of GaAs and AlAs, thus the former setting was adopted in the present work. In the cases of (GaAs)_m_(AlAs)_n_ SLs, m GaAs monolayers and n AlAs monolayers were alternately arranged along the (100) direction, as shown in Figure 1a. In the process of the geometry optimization, all of the lattice constants and positions of atoms were relaxed using the above computational methods. Based on the optimized structure of (GaAs)_m_(AlAs)_n_ SLs, the electronic structure was successfully calculated.

To get as much information regarding the (GaAs)_m_(AlAs)_n_ SLs as possible, many calculations needed to be done. In other words, we needed to run large-scale high-throughput simulation jobs for structures optimizing the simulation of the material’s properties. This situation determined a need for the management of a large number of simulation jobs, enormous quantities of data at each simulation stage, and the availability of computing resources. For this purpose, in the present work, we adopted the integrated high-throughput computational platform, MatCloud, which provided a graphical user interface for the end user to create a customized workflow for running a DFT simulation [25].

## 3. Results and Discussions

For the semiconductor superlattice, the value of the band gap is a very important parameter that determines the classification and optoelectronic performances. Figure 1b–e illustrates the band gap variation trend of (GaAs)_m_(AlAs)_n_ SLs as a function of monolayer numbers (i.e.; m and/or n). For certain thicknesses of the GaAs well as shown in Figure 1b, the band gap value of (GaAs)_m_(AlAs)_n_ SLs exponentially increased according to the function $E_{g}(n)=E_{g,GaAs}+Ae^{kn}$ (where $E_{g,GaAs}$ is band gap value of bulk GaAs, 1.508 eV, which is very close to the experimental measurements) as the thickness of AlAs barrier increases. When the monolayer number n is larger than 4, *E_g_*(n) is gradually converging to a certain value of $E_{g,w}=E_{g,GaAs}+A$. This trend is consistent in all (GaAs)_m_(AlAs)_n_ SLs, except for the values of *A* and *k*. When the AlAs barrier is ultrathin (i.e.; n < 4), the wave function of GaAs in adjacent wells could be overlapping each other, resulting in a narrowing of the band gap caused by the quantum size effect. For certain thicknesses of the AlAs barrier as shown in Figure 1c, the band gap value of (GaAs)_m_(AlAs)_n_ SLs exponentially decreases according to the function of $E_{g}(m)=E_{g,b}+Ae^{-km}$ (where $E_{g,b}+A$ is band gap value of bulk AlAs, 2.270 eV, which is also very close to the experimental measurements) as the thickness of GaAs well increases. When the monolayer number m is larger than 10, *E_g_*(m) gradually converges to a certain value of $E_{g,b}$. This trend is also consistent in all (GaAs)_m_(AlAs)_n_ SLs, except for the values of *A* and *k* (which refers to the parameters in above equations).

It is worth noticing that the fitting curves were almost identical when n was larger than 3. These results suggest that the main effect of the AlAs barrier is to impact the tunneling motion of electrons in the GaAs well caused by the quantum confinement effect. The contour map in Figure 1d is plotted using the data in the above two figures. It was found that the wide band gap of (GaAs)_m_(AlAs)_n_ SLs was concentrated in the region of m < 4, indicating the quantum size effect was outstanding in this region. When the monolayers of the GaAs well and the AlAs barrier was larger than 6, the quantum confinement effect was significant. In order to compare with the experimental measurements in published articles, the band gap values for *E_g_*(m = n) of (GaAs)_m_(AlAs)_n_ SLs (m = n) are illustrated in Figure 1e, as well as the band gap converged values of *E_g,w_*(m) and *E_g,b_*(n) in Figure 1b,c. By fitting these data, it was found that *E_g,w_*(m) exponentially decayed according to the function of $E_{g,w}(m)=E_{g,w_{0}}+Ae^{-km}$; while *E_g,b_*(n) exponentially grew according to the function of $E_{g,b}(n)=E_{g,b_{0}}-Ae^{-kn}$. The variation region of *E_g,w_*(m) was significantly larger than that of *E_g,b_*(n). In the case of (GaAs)_m_(AlAs)_n_ SLs, the value of *E_g_*(m = n) gradually decayed according to the exponential law $E_{g}(m=n)=E_{g_{0}}+Ae^{-km}$. In the latter case, the values of *E_g_*(m = n) were in food agreement with the experimental measurements in published articles [12,15,16,17,18,26,27]: *E_g_* (m = n = 1) = 2.120 eV; *E_g_* (m = n = 2) = 2.097 eV, *E_g_* (m = n = 3) = 2.099 eV, *E_g_* (m = n = 4) = 2.067 eV, *E_g_* (m = n = 5) = 2.015 eV, *E_g_* (m = n = 7) = 1.918 eV, *E_g_* (m = n = 8) = 1.881 eV, and *E_g_* (m = n = 10) = 1.766 eV. For example, Fujimoto et al. found the band gaps of (GaAs)_n_(AlAs)_n_ SLs with n = 1–15 varied according to the exponential decay with increasing period number [12], which is consistent with the experimental measurements by Jiang et al. [27] and our finding in the present work. However, for the specific numerical value, the reported band gap values are also inconsistent in published articles. For instance, Barkissy et al. measured the band gap energy of GaAs (d = 28.3 Å)/AlAs (d = 28.3 Å) SL as 1.748 eV [15], which is close to our calculated result (the band gap of (GaAs)_5_(AlAs)_5_ SL was 1.684 eV); Fujimoto et al. also measured the band gap energy of (GaAs)_5_(AlAs)_5_ SL as 1.910 eV through photoluminescence measurements at room temperature [12]. The difference between experimental measurements and theoretical calculations is ascribed to the measurement error, temperature effects, theoretical approximation, and so on.

**Figure 2:**
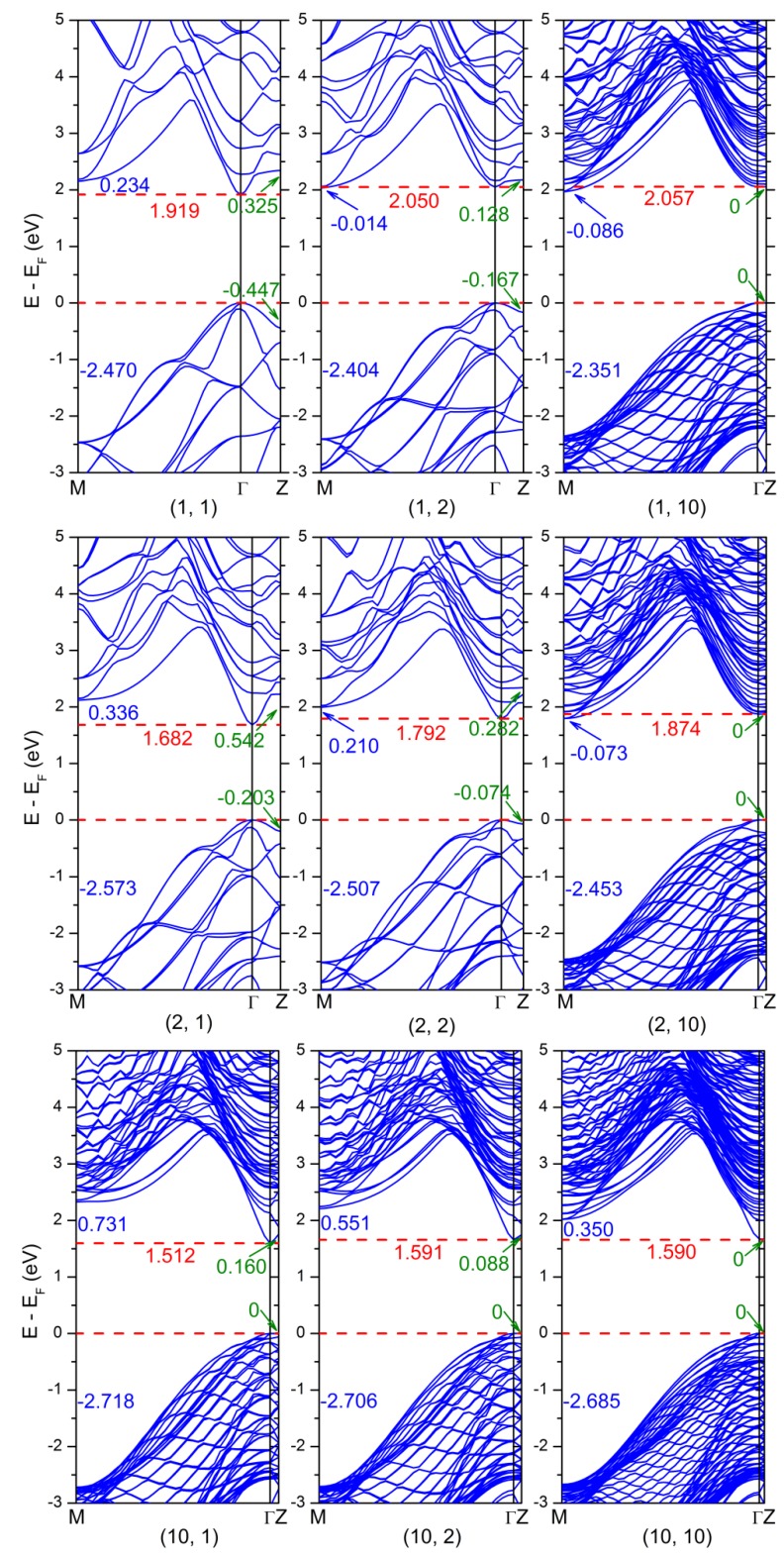
The calculated band structure of nine representative (GaAs)_m_(AlAs)_n_ SLs (m, n = 1, 2, 10). The numerical values denoted by different-colored fonts refer to the energy eigenvalues of the upper valence band at the Γ-point.

To deeply explore the detailed variation of the band gap, the typical band structure of (GaAs)_m_(AlAs)_n_ SLs are shown in Figure 2, in which the numerical values denoted by different-colored fonts refer to the energy eigenvalue of the upper valence band at Γ-point. Several key characteristics are summarized as follows: (1) When the thickness of the AlAs barrier is increasing for a certain thickness of the GaAs well, the energy eigenvalue of the lower conduction band at Γ-point (Γ_CB_) significantly increased, while the energy eigenvalue of the lower conduction band at M-point (M_CB_) was slightly decreasing. On the contrary, the energy eigenvalue of the upper valence band at the M-point (M_VB_) increased significantly. (2) When the thickness of the GaAs well was increasing for a specified thickness of the AlAs barrier, both Γ_CB_ and M_VB_ decreased significantly, while M_CB_ increased significantly. (3) As the period of the SLs was increasing, the energy eigenvalue of the lower conduction band at Z-point (Z_CB_) was gradually decreasing, while the energy eigenvalue of the upper valence band at Z-point (Z_VB_) was gradually increasing. Finally, the values of Z_CB_ and Z_VB_ were identical with that of Γ_CB_ and Γ_VB_, respectively, meaning that the quantum confinement effect gradually predominated.

These variations of the band structure of (GaAs)_m_(AlAs)_n_ SLs led to two important phenomena: the first is the variations of the band gap as shown in Figure 1, and the second is the direct-to-indirect-gap transition. In the case of a (GaAs)_1_(AlAs)_1_ SL, the band gap is a Γ-Γ direct band gap. With a decrease of M_CB_, the cases of (GaAs)_1_(AlAs)_n_ SLs (n ≥ 2) were a Γ-M indirect band gap owing to the crossover between Γ_CB_ and M_CB_. In the case of a (GaAs)_2_(AlAs)_4_ SL, the value of M_CB_ was almost equal to the value of Γ_CB_, thus (GaAs)_2_(AlAs)_n_ SLs (n < 4) were Γ-Γ direct band gaps, while (GaAs)_2_(AlAs)_n_ SLs (n > 4) had Γ-M indirect band gaps by the same underlying mechanism. These calculated results are very consistent with previous reported experimental measurements [15,18,28,29], showing that a crossover in the band structures of (GaAs)_m_(AlAs)_n_ SLs with m = n takes place in a certain range of the layer thickness at low temperatures, resulting in an indirect–direct band gap transition. As known, bulk GaAs is a direct band gap semiconductor with *E_g_* = 1.508 eV, while bulk AlAs is an indirect band gap semiconductor with *E_g_* = 2.270 eV. Based on the above data analysis, it can be concluded that: (1) When the periodic number of the GaAs well or AlAs barrier was small (i.e. the thickness was small), the quantum confinement effects of the GaAs well and AlAs barrier were affected and coupled with each other, and the quantum confinement effect of the GaAs well was predominant, resulting in the direct band gap of (GaAs)_m_(AlAs)_n_ SLs. (2) When the periodic number of the GaAs well or AlAs barrier was large (i.e. the thickness was large), the quantum confinement effect coupling between the GaAs well and AlAs barrier gradually weakened and decoupled, thus the band gap of (GaAs)_m_(AlAs)_n_ SLs were determined by the AlAs barrier, resulting in the indirect band gap of (GaAs)_m_(AlAs)_n_ SLs. (3) The thickness of the AlAs barrier had a stronger impact on the electronic properties of (GaAs)_m_(AlAs)_n_ SLs. In the present work, (GaAs)_m_(AlAs)_n_ SLs (m >3, n ≤ 10) were all Γ-Γ direct band gaps. Due to the limitations of computational resources, the larger size of the (GaAs)_m_(AlAs)_n_ SLs (m, n > 10) were not considered. However, it could be predicted that (GaAs)_m_(AlAs)_n_ SLs will transform from a direct band gap to an indirect band gap when the AlAs barrier is thick enough, according to above band gap variation and laws.

**Figure 3:**
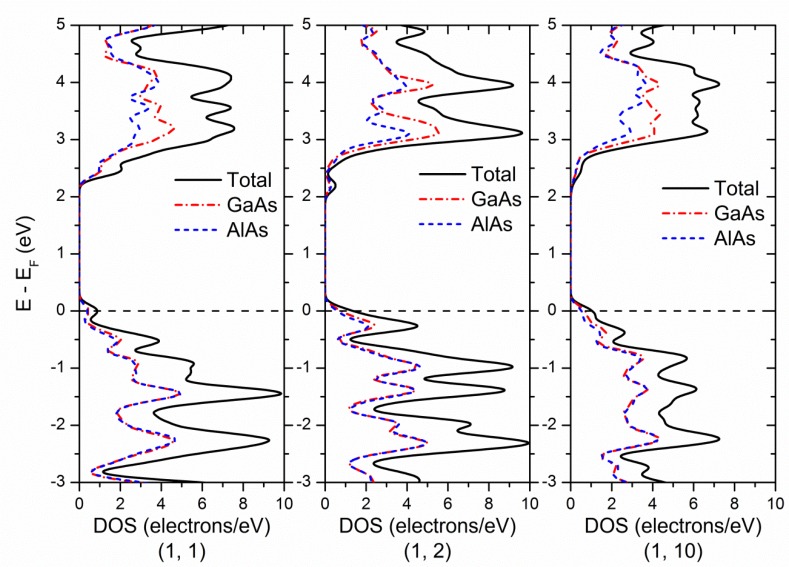
The calculated total and local density of states of (GaAs)_1_(AlAs)_n_ SLs (n = 1, 2, 10).

In order to explain the nature of the above trends, the local density of states of (GaAs)_1_(AlAs)_n_ SLs (n = 1, 2, 10) are provided and analyzed in Figure 3. Compared to the local partial density of states of GaAs and AlAl bulk (not showed here), it was found that M_CB_ was mainly contributed by the Al 3p states, and Γ_CB_ was mainly contributed by the hybridized states between Ga 4s and 4p states. Thus, in the case of (GaAs)_m_(AlAs)_n_ SLs, as the thickness of the AlAs barrier increased, the contribution of the Al 4p states for the lower conduction band gradually decreased and relatively down-shifted, while the contribution of hybridized states between Ga 4s and 4p states gradually decreased and relatively up-shifted. This was the main reason for the (GaAs)_m_(AlAs)_n_ SLs transformation from having a Γ-Γ direct band gap to having a Γ-M indirect band gap. Furthermore, above variation trends can be explained using the classic semiconductor theory: when the thickness of a GaAs well is constant, the interaction between adjacent GaAs wells is gradually weakened with the increase of the thickness of the AlAs barrier, so the contribution of the AlAs electronic states is gradually enhanced; when the thickness of the AlAs barrier is constant, the quantum size effect is gradually weakened and the contribution of the GaAs electronic states is gradually enhanced with the increase of the thickness of the GaAs well.

**Figure 4:**
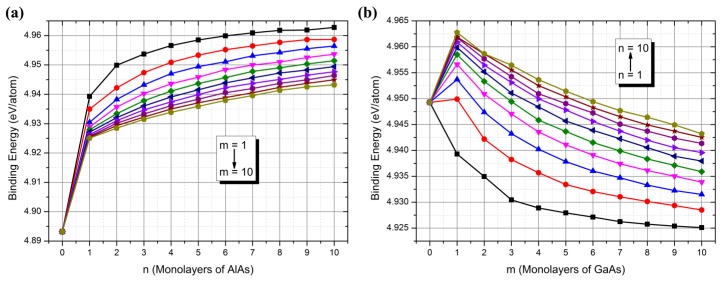
The calculated binding energy of (GaAs)_m_(AlAs)_n_ SLs (m, n ≤ 10) as a function of the number of monolayers of the GaAs well or AlAs barrier.

To examine the stability of the whole system, the binding energy (*E_b_*) was further investigated. The binding energy was calculated using the following equation: $E_{b}=\left[E_{SLs}-\sum_{i}n_{i}E_{i}\right]/\sum_{i}n_{i}$, where *E_SLs_* is the total energy of the (GaAs)_m_(AlAs)_n_ SLs, and *n_i_* and *E_i_* are the number and energy of the isolated *i* atom (Ga, Al, or As), respectively. As shown in Figure 4, we found another interesting phenomenon in the present work. The variation trends of the binding energy of (GaAs)_m_(AlAs)_n_ SLs were very consistent with those of the band gap as shown in Figure 1b,c. For the GaAs well, the introduction of the AlAs barrier could increase the binding energy, indicating the stability of (GaAs)_m_(AlAs)_n_ SLs was better than that of the GaAs bulk material. For the AlAs barrier, the introduction of a few GaAs wells could increase the binding energy in the cases of (GaAs)_m_(AlAs)_n_ SLs (m < 5, n > 3), while in the other cases, the stability of the (GaAs)_m_(AlAs)_n_ SLs was worse than that of the AlAs bulk material. Importantly, owing to the identical variation trends between the binding energy and the band gap, it indicated that the binding energy could be used as a detector to estimate the band gap value in the design of (GaAs)_m_(AlAs)_n_ devices.

## 4. Conclusions

In summary, using a high-throughput study based on DFT calculations, the electronic structure and properties of short-period (GaAs)_m_(AlAs)_n_ (m, n ≤ 10) superlattices were thoroughly investigated and systematically compared. For certain thicknesses of the GaAs well, the band gap value of (GaAs)_m_(AlAs)_n_ SLs exponentially increased, while for certain thicknesses of the AlAs barrier, the band gap value of (GaAs)_m_(AlAs)_n_ SLs exponentially decreased. In both cases, the band gap values converged to certain values. However, these converged values varied by different laws as a function the thickness of the GaAs well or AlAs barrier. Furthermore, owing to the energy eigenvalues at different k-points showing different variation trends, (GaAs)_m_(AlAs)_n_ SLs transformed from having a Γ-Γ direct band gap to having a Γ-M indirect band gap when AlAs barrier was thick enough. The intrinsic reason for these variations was due to the contributions and positions of the electronic states of the GaAs well or AlAs barrier having changed under different thickness conditions. Moreover, it was found that the binding energy could be used as a detector to estimate the band gap value in the design of (GaAs)_m_(AlAs)_n_ devices.
